# Supplementary material for: BH3 mimetics targeting BCL-XL have efficacy in solid tumors with RB1 loss and replication stress
Source: Nat Commun. 2025 May 28;16:4931. doi: 10.1038/s41467-025-60238-x (PMC12119881; doi:10.1038/s41467-025-60238-x)
Supplement: Supplementary file 1 — Supplementary Information [file 41467_2025_60238_MOESM1_ESM.pdf]

Model		Maximum Responses (% reduction from control)			IC50 Navitoclax (nM)	IC50 S63845 (nM)
		Navitoclax	Venetoclax	S63845		
LuCaP Primary Cultures	147	64	12	0	300	-
	176	78	3	4	300	-
	78	66	10	0	750	-
	23.1	50	17	10	1000	-
	136CR	33	6	2	-	-
	189.4	26	24	14	-	-
	235.3	29	2	5	-	-
3D Organoids	BIDPC1	60	9	0	125	-
	BIDPC4	7	10	13	-	-
	BIDPC5	90	3	12	100	-
	BIDPC6	70	23	53	250	500
	BIDPC7	94	21	6	<50	-
	15-442-12	74	16	38	250	-
	LuCaP35CR	10	5	80	-	40
	LuCaP70CR	20	25	90	-	50
	LuCaP77	55	12	16	400	-
Cell Lines	VCaP	5	5	90	-	75
	LNCaP	5	0	6	-	-
	C42	17	0	5	-	-
	DU145	0	0	3	-	-
	PC3	7	0	5	-	-

Supplementary Table S1. BH3 mimetic screening in PCa organoids and cultures. Maximum response and IC50 are shown.

Drug	Target	Category						
		TKI	CDK	Spindle	PDK	MDM2	PLK1	Other
UK356618	matrix metalloprotease-3							other
Ascomycin	antibiotics							other
kbNB 14270	selective protein kinase D (PKD) inhibitor	TKI						
FK866	nicotinamide phosphoribosyltransferase							other
SP2509	histone demethylase LSD1 inhibitor							other
AS1517499	STAT6 inhibitor	TKI						
Defactinib	ocal adhesion kinase (FAK) inhibitor	TKI						
MI-773	MDM2 antagonist					MDM2		
YK4279	EWS-FLI1 binding to RNA helicase A (RHA).							other
RO495	Non-receptor tyrosine-protein kinase 2 (TYK2	TKI						
SB225002	CXCR2 Antagonist							other
PD176252	non-peptide gastrin-releasing peptide receptor (GRP-R, BB2)							other
MK886	5-lipoxygenase-activating protein (FLAP)							other
PHA 767491	CDK9, CDK7		CDK					
PF514273	CB1 receptor antagonist							other
GSK923295	allosteric inhibitor of CENP-E kinesin motor ATPase			Kinesin				
PF3758309	TP-competitive, pyrrolopyrazole inhibitor of PAK4	TKI						
BX912	ATP-Competitive PDK1 Inhibitor.				PDK1			
GSK2334470	PDK1 Inhibitor				PDK1			
MG-132	proteasome inhibitor							other
KPT-330	Exportin-1 (XPO1)							other
Eltrombopag	thrombopoetin							0ther
AZD8055	mTOR kinase inhibitor	TKI						
Volasertib	PLK1 inhibitor						PLK1	
BMN-673	PARP1-2 inhibitor							other
CP-673451	PDGFR inhibitor	TKI						
OTSSP167	MELK-selective inhibito							other
Dihydroartemisinin	artemisinin component/ free radicals							other
BAY61-3606	Syk inhibitor	TKI						
Ispinesib	kinesin spindle protein			kinesin				
Obatoclax	BCL2, BCLXL, MCL1							other
Nolatrexed	Thymidine Synthase inhibitor							other
Papaverine	phosphodiesterase							other
BI2536	PLK1 PLK4						PLK1	
L-779450	B-Raf kinase inhibitor	TKI						
LY2090314	GSK-3 inhibitor							other
CCT241533	CHK2 inhibitor	TKI						
UNC2881	Mer tyrosine kinase inhibitor	TKI						
MBX-2982	GPR119 agonist							other
Ro-3306	CDK1 inhibitor		CDK					
UNC0631	Protein lysine methyltransferase G9a							other
BNC105	tubulin polymerization inhibitor			tubulin				
RG7388	MDM2 inhibitor					MDM2		
MK2206	allosteric Akt inhibitor							other
Nutlin	MDM2 inhibitor					MDM2		
ARRY520	kinesin spindle protein (KSP) inhibitor			kinesin				
Rupatadine	H1 histamine, PAFR antagonist							other
PF-431396	FAK and PYK2 inhibitor	TKI						
GSK126	EZH2 methyltransferase inhibitor							other
AZD6482	PI3Kβ inhibitor	TKI						
SB525334	PI3Kβ-selective inhibitor	TKI						
CH5132799	pan-PI3 kinase inhibitor	TKI						
AZD5438	CDK 1.2.9		CDK					
AZ20	ATR inhibitor	TKI						
Rimonabant	cannabinoid receptor CB1							other
Abemaciclib	CDK4/6		CDK					
Spautin-1	Beclin-1 degrater, autophaty							other
CCT128930	Akt inhibitor	TKI						
AXL1717	IGF1R inhibitor	TKI						
SB-743921	kinesin spindle protein			kinesin				
Epothilone	microtubule-stabilizing agents			microtubules				
TAK960	PLK1						PLK1	
AZD1332	neurotrophic tyrosine kinase receptors antagonist	TKI						
Differine	naphthoic acid							other
PG01	CFTR Cl- channel potentiator							other
BIP135	glycogen synthase kinase-3							other
Rotenone	Mitochondrial Complex I Inhibitor							other
BRD6929	HDAC1 and HDAC2 inhibitor							other
JNJ10198409	PDGFR inhibitor	TKI						
Mozavaptan	vasopressing receptor antagonist							other
		20	4	6	2	3	3	32

Supplementary Table S2. Agents that enhancd responses to navitoclax in primary drug screen.

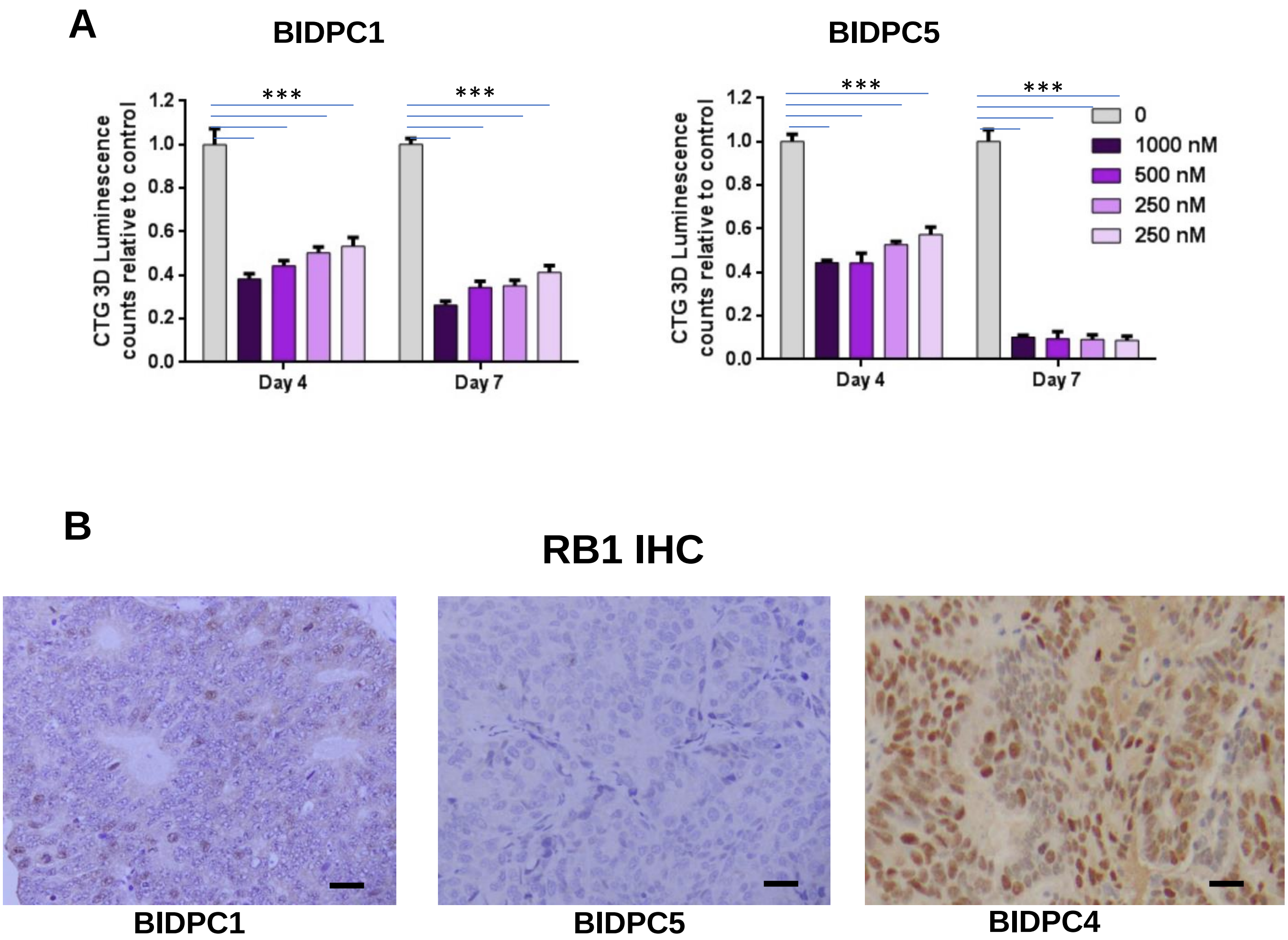

Supplementary Figure S1. (A) Sustained navitoclax responses in PCa organoids with longer culture duration. Primary organoid cultures from BIDPC1 or BIDPC5 PDXs were treated for 4 or 7 days with navitoclax at the indicated concentrations and assessed for caspase activation (Caspase Glo 3/7). Data show mean values and SEM from biological triplicates. One way ANOVA test was performed for comparison *** $p<0.001$. (**B)** FFPE blocks from untreated BIDPC1, 4, and 5 PDX were stained for RB1 (clone 4H1). Antibody dilution was 1:2000. Weak staining in subset of cells in BIDPC1 may be nonspecific or reflect small subset of positive cells. Scale bar is 20 μM.

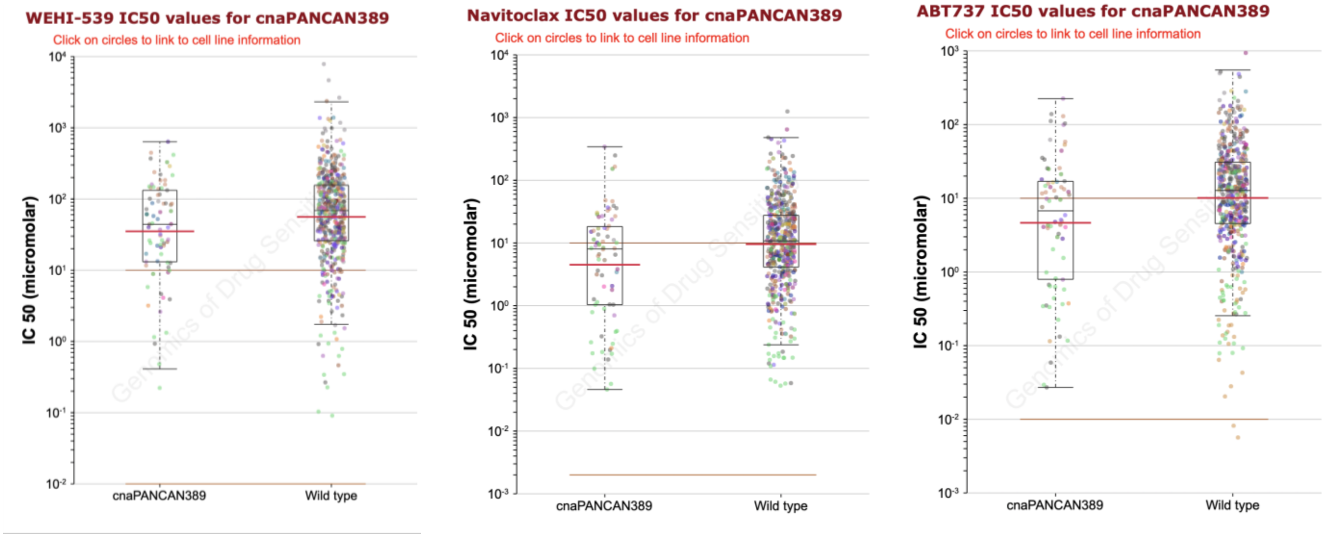

Supplementary Figure S2. RB1 loss sensitizes to navitoclax. Sanger data on IC50 for BH3 mimetics in solid tumor cells with *RB1* copy number loss (cnaPANCAN389).

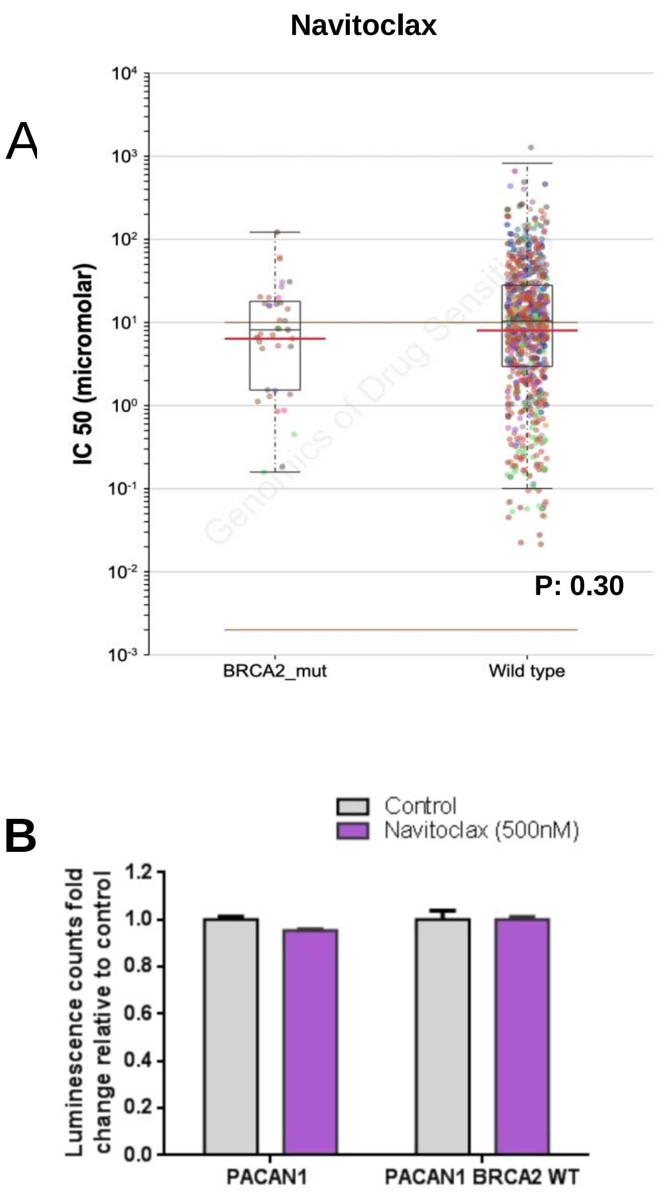

Supplementary Figure S3. BRCA2 loss does not sensitize to navitoclax. **(A)** Sanger data on IC50 for navitoclax in BRCA2 altered versus wild-type cells. Analysis by one way ANOVA indicated the difference was not significant (p=0.30). **(B)** Effect of navitoclax in BRCA2 deficient PANCAN1 cells and in an isogenic line with restored BRCA2. Cells were treated for 48 hrs with 500 nM navitoclax or vehicle control (DMSO). Data are mean and SEM from biological triplicates.

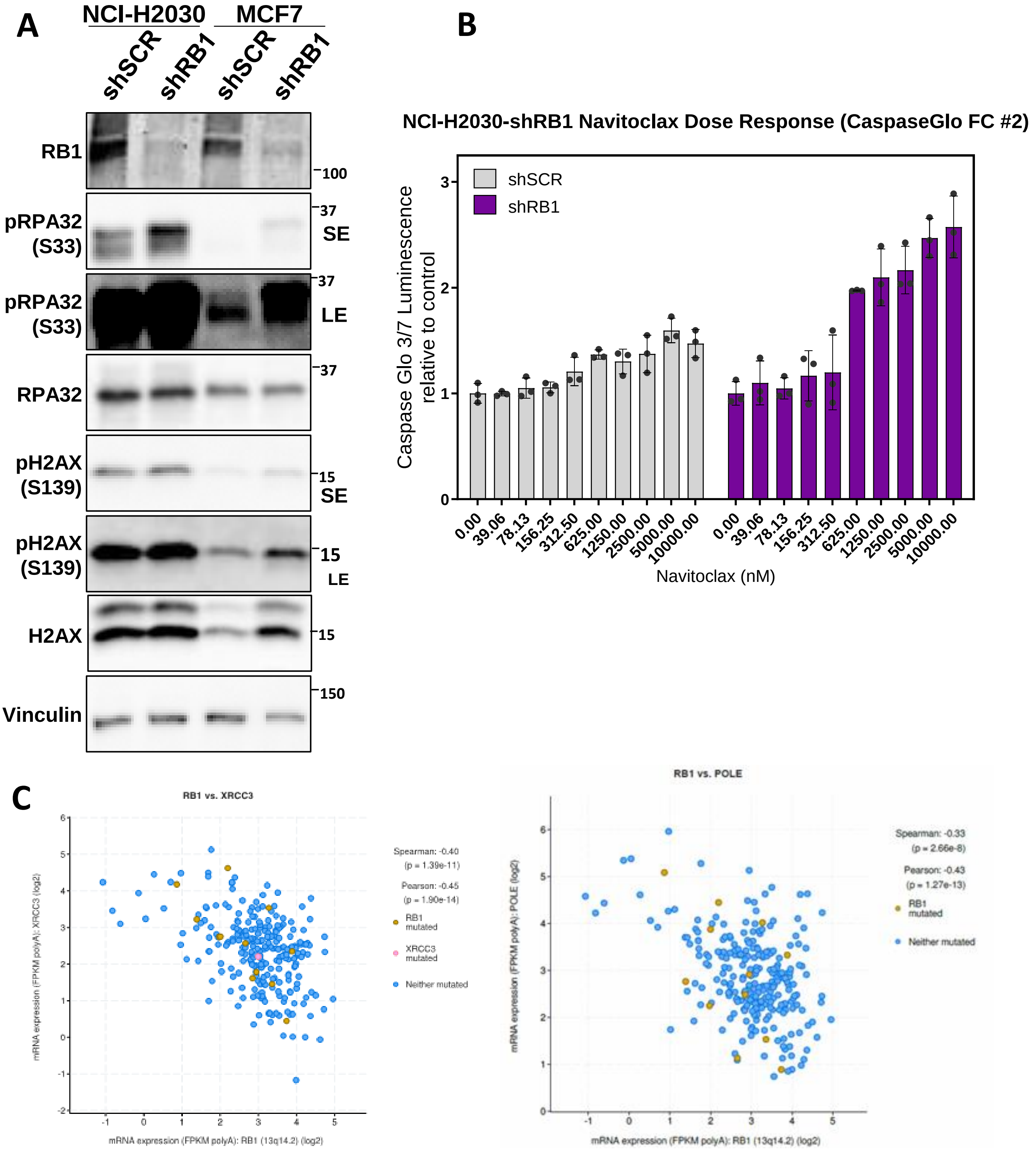

Supplementary Figure S4. RB1 loss increases DNA damage. (A) NCI-H2030 and MCF7 cells expressing doxycycline-regulated RB1 or scrambled control shRNA were treated with doxycycline (500 ng/ml) for 3 days to induce shRNA and then lysed and assessed by immunoblotting as indicated. Short (SE) and long (LE) exposures are shown for some proteins. **(B)** NCI-2030 cells expressing doxycyline-induced RB1 or control shRNA were treated with navitoclax for 24 hrs and assessed for caspase activation. Data are mean and SEM of biological triplicates, and are normalized to the respective no navitoclax controls. Analysis by two way ANOVA showed that the RB1 shRNA significantly increased levels of Caspase Glo activity in response to navitoclax (p<0.001). **(C)** DNA repair gene and RB1 expression are negatively correlated in primary PCa. XRCC3 and POLE expression were correlated with RB1 expression in TCGA primary PCa (data from cBioportal).

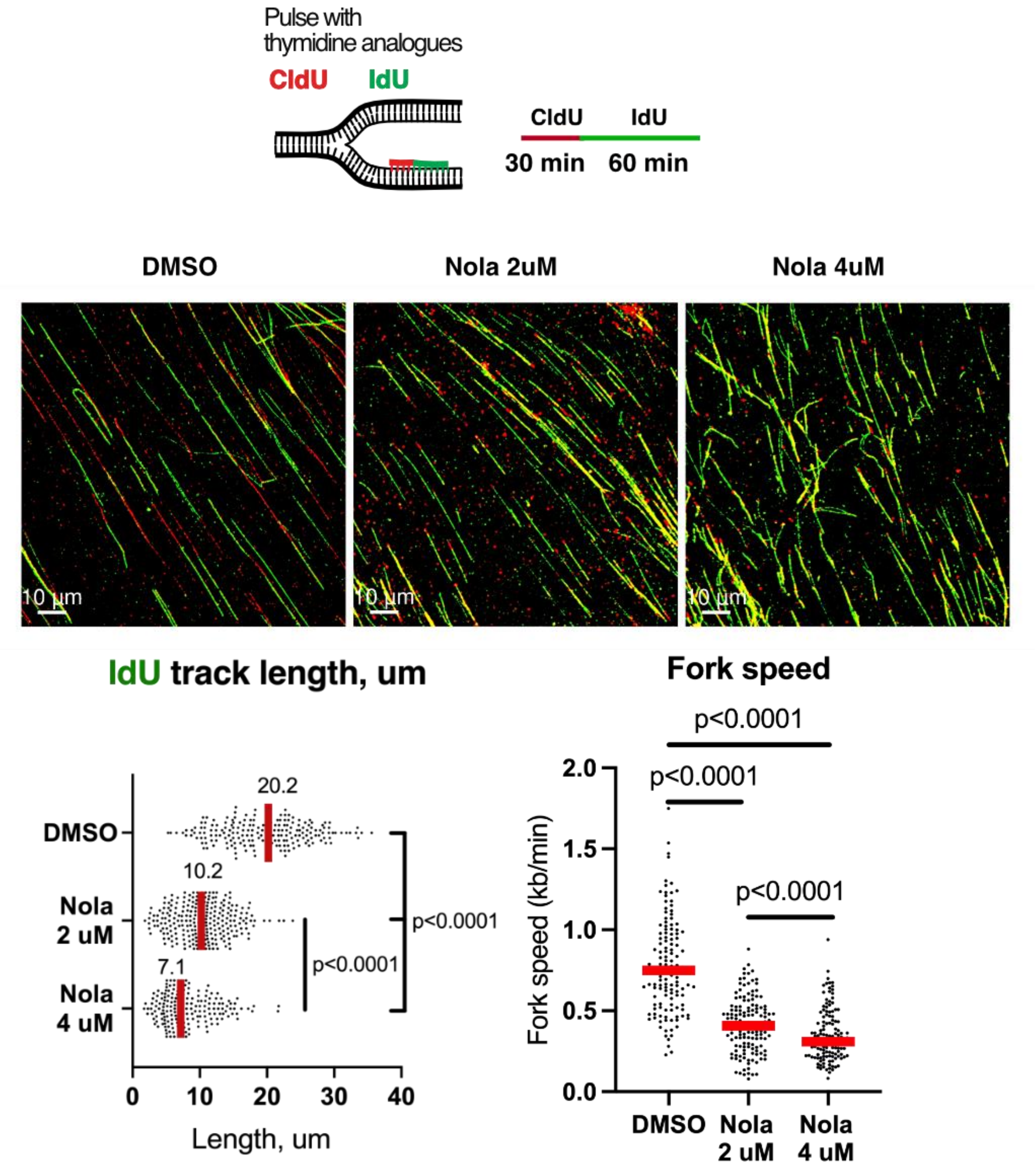

Supplementary Figure S5. Replication fork dynamics were analysed by DNA Fiber Assay. LNCaP cells were treated with either DMSO or nolatrexed (2 µM or 4 µM) for 16h. Cells were labelled with CldU followed by IdU as indicated. Representative images are shown, scale bar: 10 µm. Median IdU track lengths (µm) of at least 200 double-labelled fibers are shown in red. Mann–Whitney test was applied to test significance. Replication fork speed was calculated using conversion factor 1 µm=2.59 kb. Values were plotted and median fork speed is shown in red for each treatment condition. Mann–Whitney test was applied to test significance.

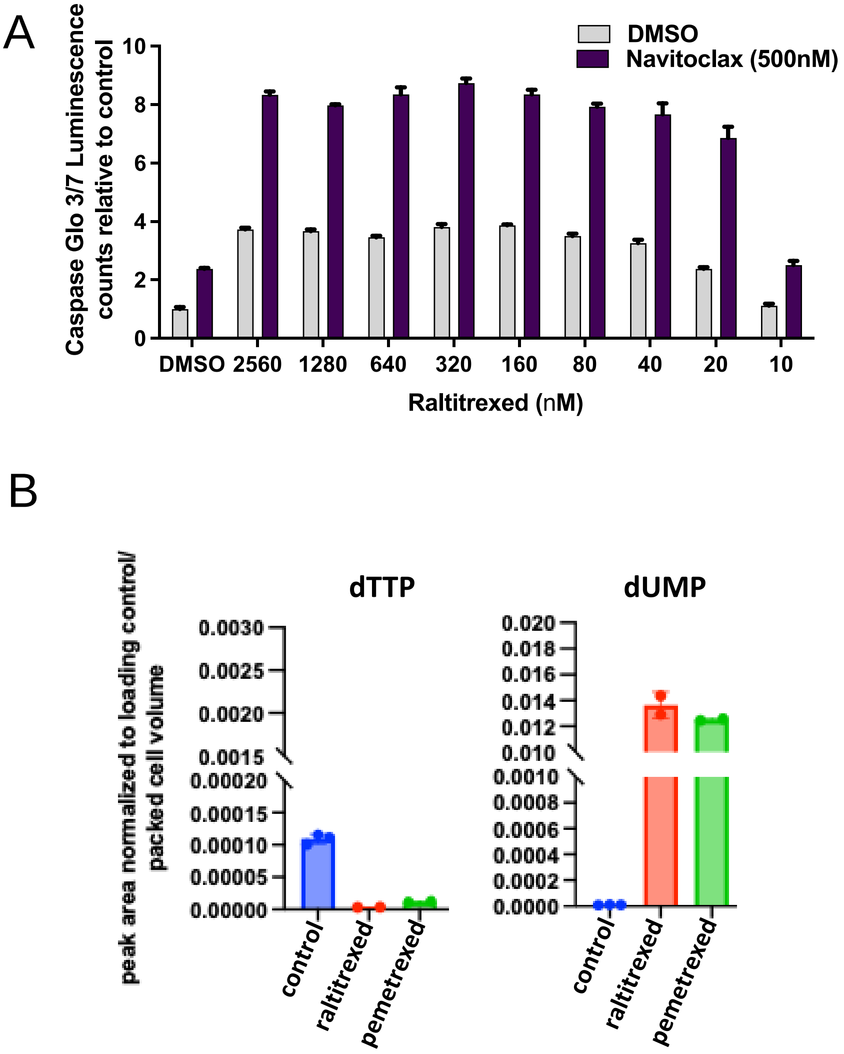

Supplementary Figure S6. Nucleotide depletion due to thymidylate synthase inhibition correlates with sensitization to navitoclax. (A) LNCaP cells were treated with raltitrexed for 48 hours followed by 6 hours treatment with navitoclax as indicated. Apoptosis was then assessed by Caspase Glo assay. Data shown are mean and SEM from biological triplicates and are normalized to cells that only got vehicle. Analysis by two way ANOVA showed that raltitrexed significantly enhanced the apoptotic response to navitoclax ($p<0.001$). **(B)** Levels of dTTP and dUMP were assessed by mass spectrometry in LNCaP cells treated for 24 hours with 100 nM raltitrexed or pemetrexed. Data are mean and SEM of biological triplicate samples.

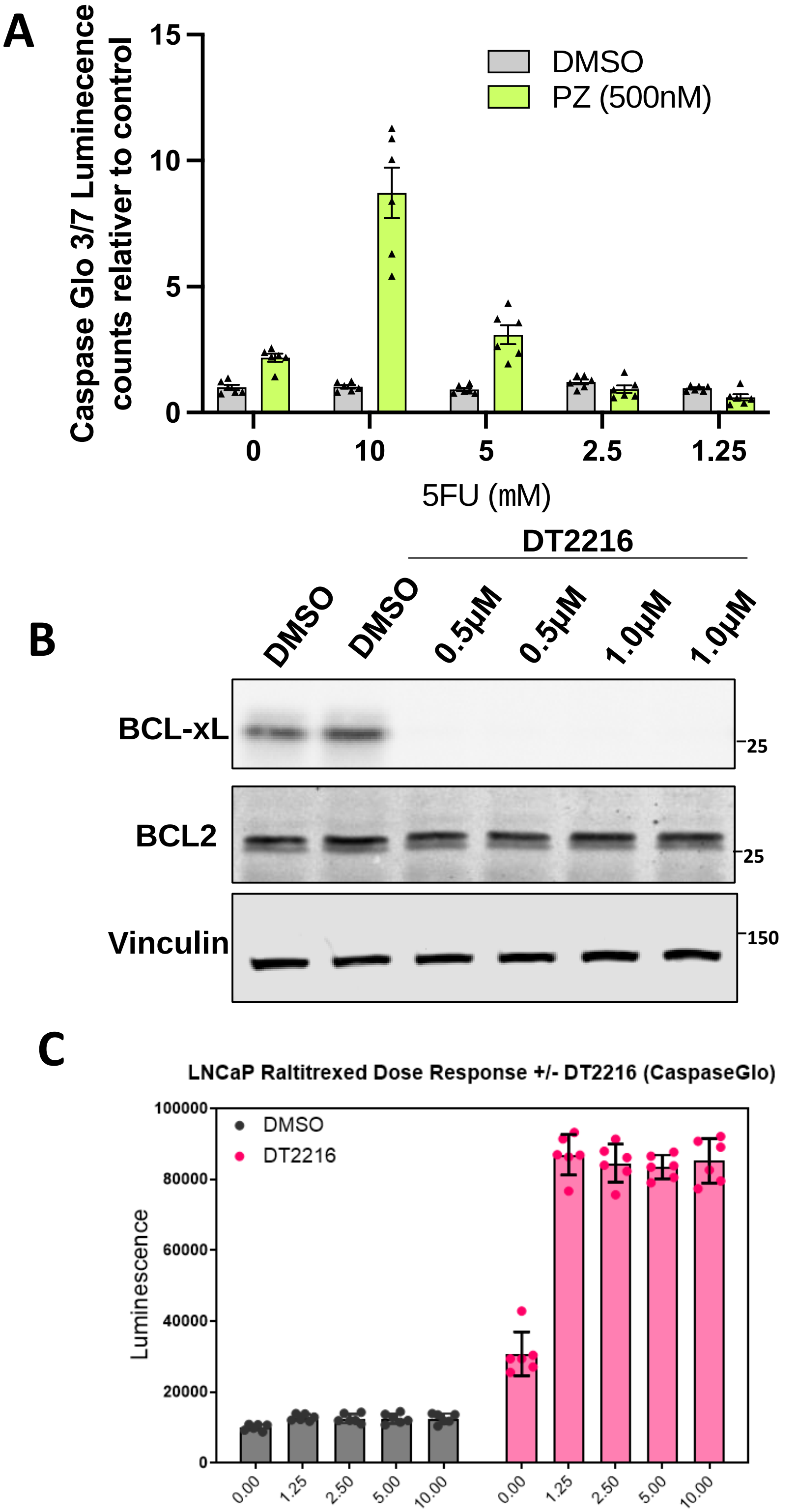

Supplementary Figure S7. Cooperativity between thymidylate synthase inhibition and BCL-XL degraders. (A) LNCaP cells were treated for 48 hours with 5-FU followed by PZ18755B (BCL2/BCL-XL degrader) for 16 hours, and apoptosis was assessed by Caspase Glo 3/7. Data are mean and SEM of biological replicates (n=6). Analysis by two way ANOVA showed that 5FU significantly enhanced the apoptotic response to navitoclax ($p<0.001$). **(B)** LNCaP cells were treated for 16 hours with DT2216 (BCL-XL selective degrader) and assessed by immunoblotting. (**C,**) LNCaP cells were treated for 48 hours with raltitrexed followed by 16 hours with DT2216 and Caspase Glo assay. Data are mean and SEM of biological replicates (n=6). Analysis by two way ANOVA showed that raltitrexed significantly enhanced the apoptotic response to navitoclax ($p<0.001$).

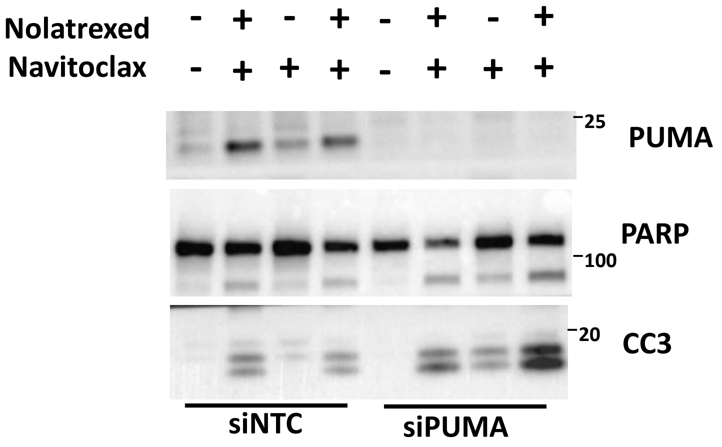

Supplementary Figure S8. Nolatexed sensitization to navitoclax is not dependent on PUMA. LNCaP cells were treated with PUMA or nontarget control siRNA for 3 days in total. During last 48 hours they were treated with nolatrexed (500 nM), and during last 6 hours with navitoclax (500 nM).

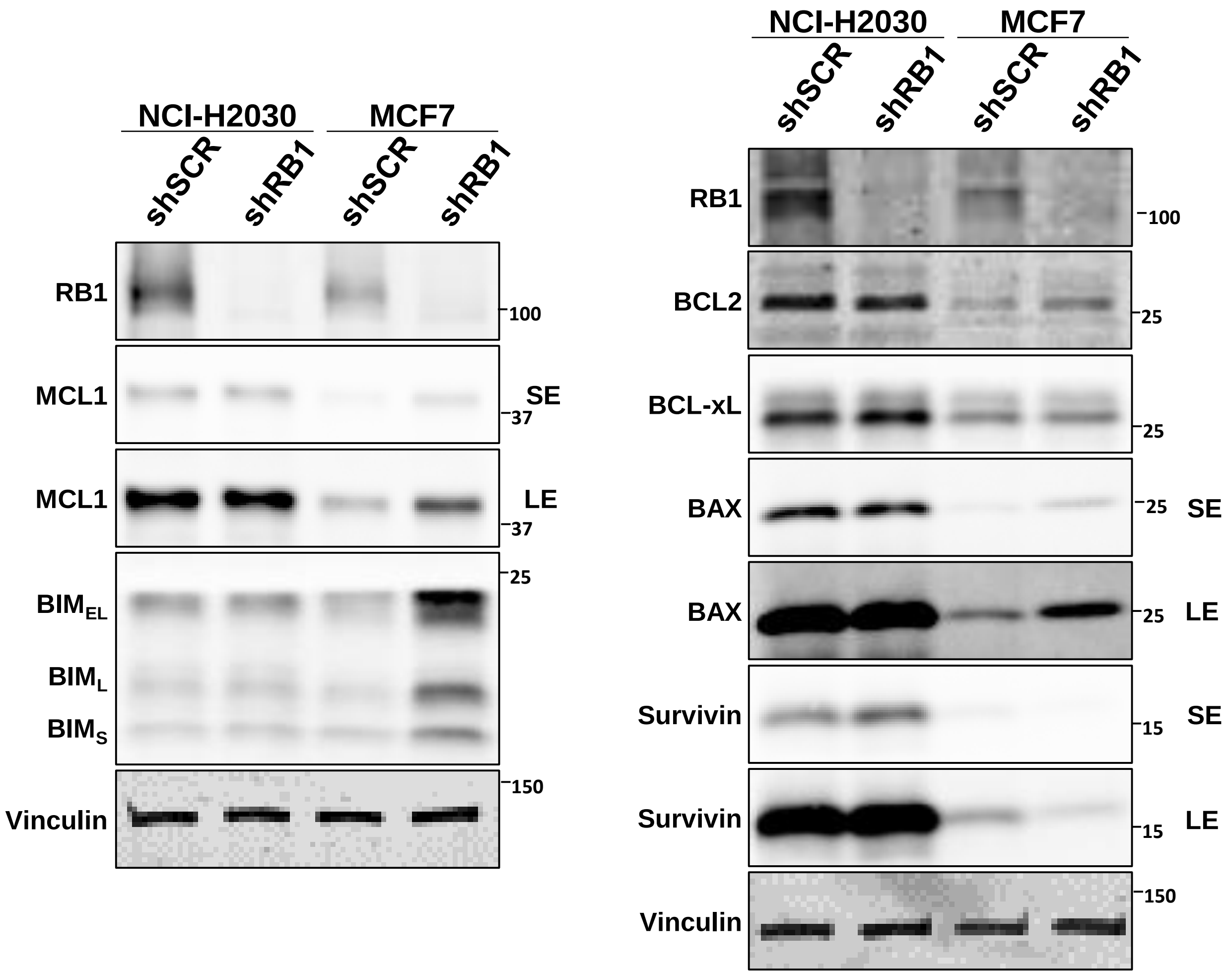

Supplementary Figure S9. Effects of RB1 downregulation on apoptosis-related proteins. NCI-H2030 and MCF7 cells expressing RB1 or scrambled control shRNA were lysed and assessed by immunoblotting as indicated. Short (SE) and long (LE) exposures are shown for some proteins.

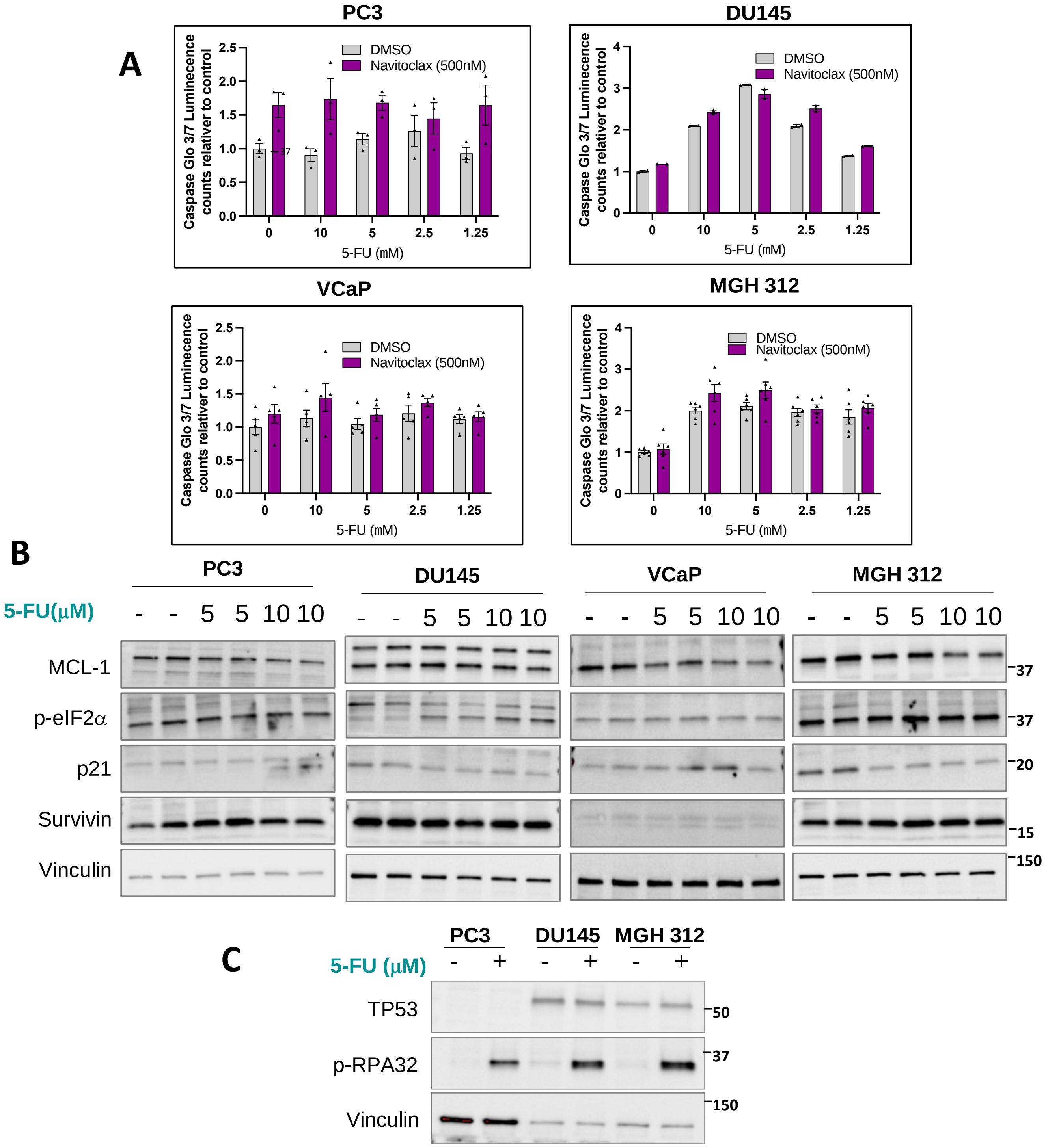

Supplementary Figure S10. Thymidylate synthase sensitization to BCL-XL inhibition is p53 dependent. (A) TP53 mutant solid tumor cell lines (PC3-prostate cancer, DU145-prostate cancer, VCaP-prostate cancer, MGH312-breast cancer) were treated with the combination of 5-FU (48 hours) and navitoclax (added for last 6 hours). Caspase 3/7 activity was assessed with immunofluorescence-based assay. Data are mean and SEM of biological replicates (n=5). (**B**) Immunoblotting analysis for p53, integrated stress response, and survivin in TP53 deficient cell lines treated with 5-FU for 48 hours. **(C)** Immunoblotting for replication stress marker (pRPA32) in TP53 deficient cell lines treated with 5-FU for 48 hours.

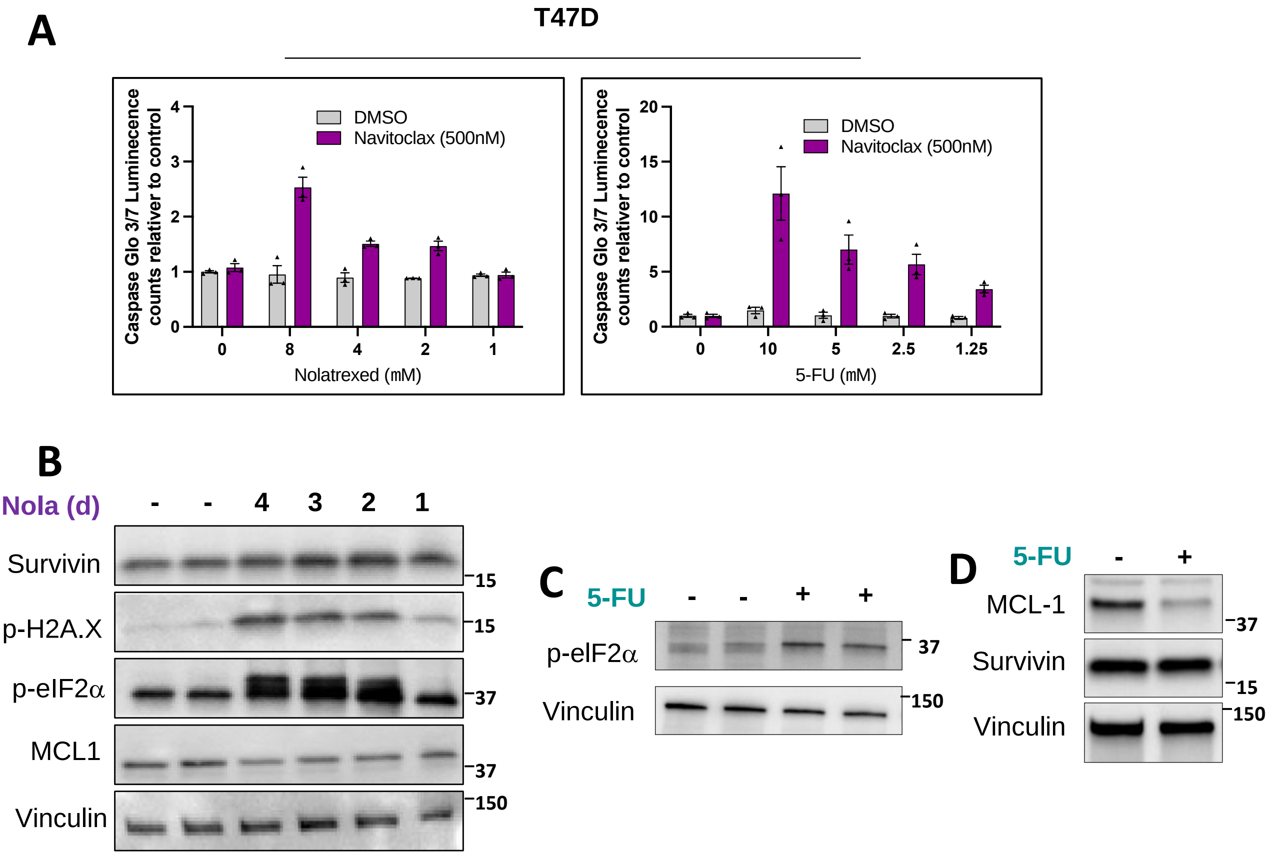

Supplementary Figure S11. Thymidylate synthase inhibition sensitization to BCL-XL inhibition in T47D through activation of an integrated stress response. (**A)** Caspase 3/7 activity in T47D (TP53 deficient breast cancer) cell line treated for 48 hours with the combination of nolatrexed plus navitoclax (left) and 5-FU plus navitoclax (right). Data are mean and SEM of biological triplicates. Analysis by two way ANOVA showed that both nolatrexed and 5-FU significantly enhanced the apoptotic response to navitoclax ($p<0.001$). (**B)** Immunoblotting analysis of integrated stress response marker (p-eIF2α) and apoptotic machinery proteins in T47D cells treated with nolatrexed (2 μM) for 1-4 days. (**C, D)** T47 cells were treated with 5-FU for 48 hours. Immunoblotting analysis was performed for integrated stress response marker p-eIF2α (C) or for MCL-1 and survivin (D).

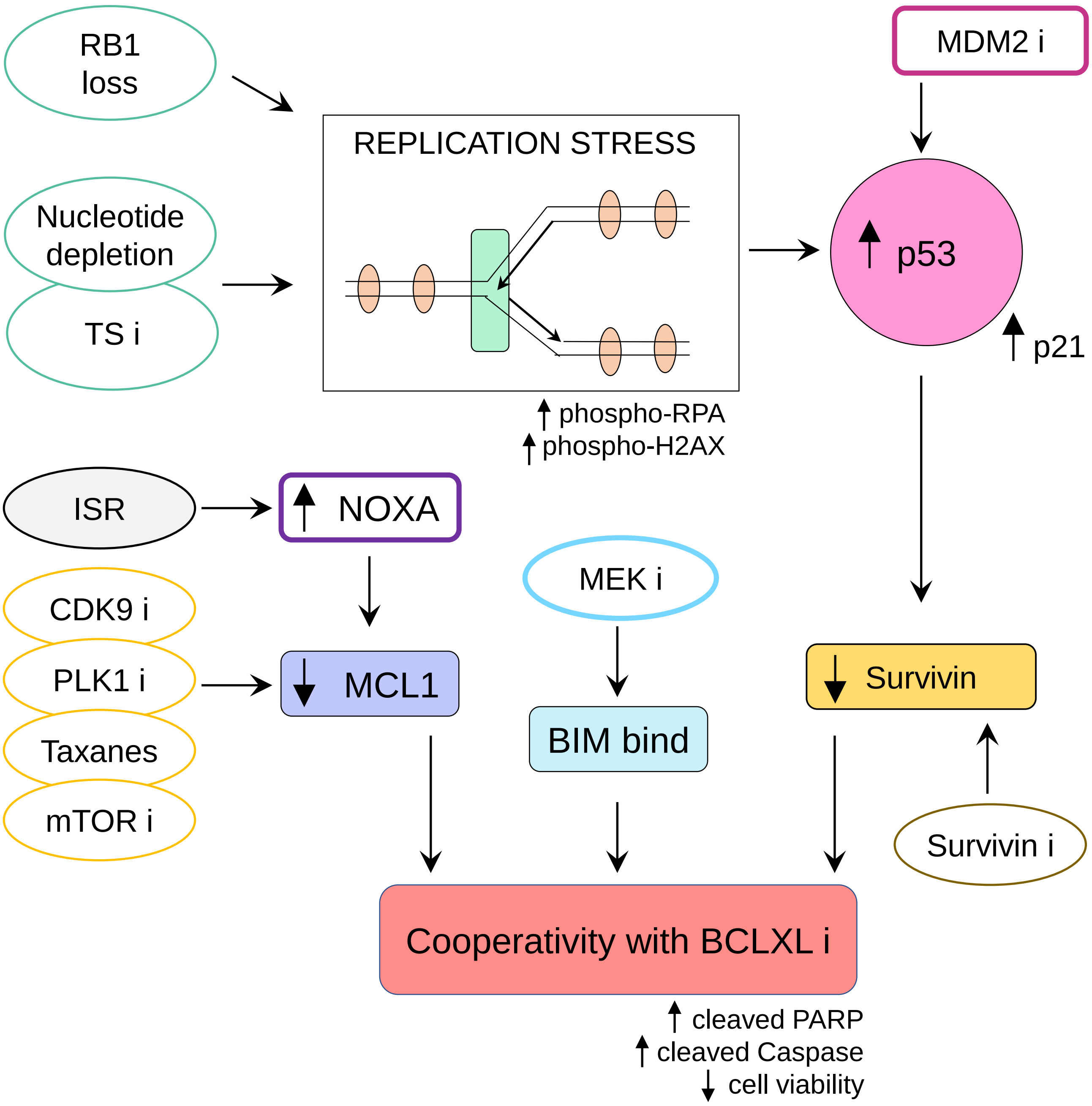

Supplementary Figure S12. Overview of mechanisms that increase dependence on BCL-XL. One group of mechanisms converges on MCL1 to reduce its expression or increase its degradation. A second group described here converges on survivin. Survivin expression is also cell cycle regulated and its activity may be modulated by phosphorylation (not shown).

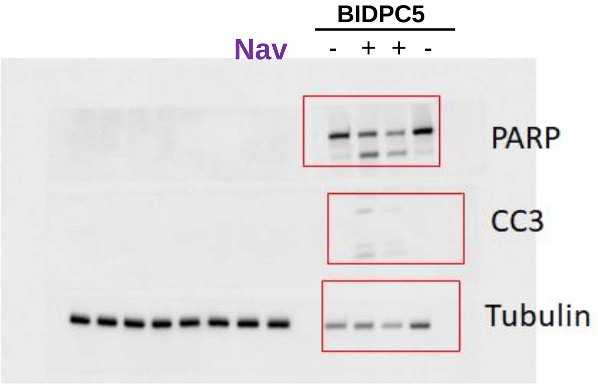

Supplementary Figure S13. Uncropped gels related to Figure 1

E

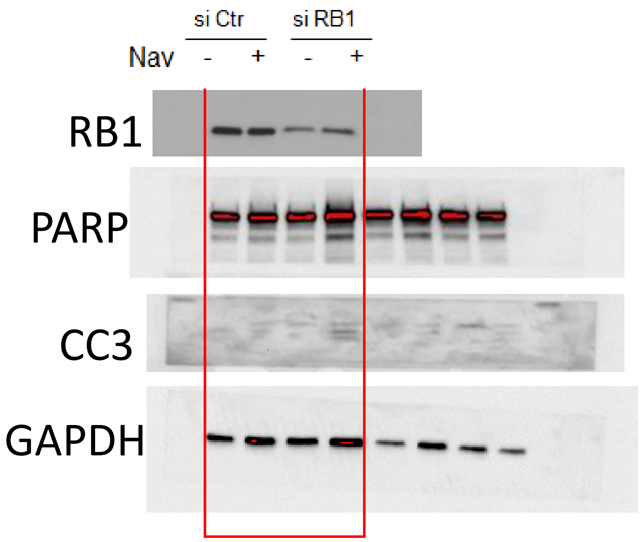

F

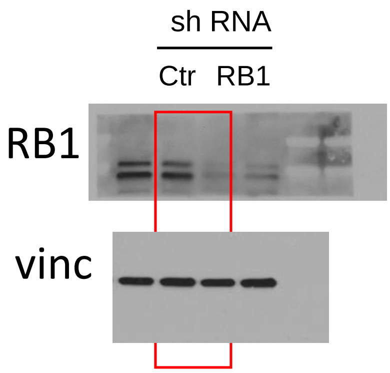

Supplementary Figure S14. Uncropped gels related to Figure 2.

E

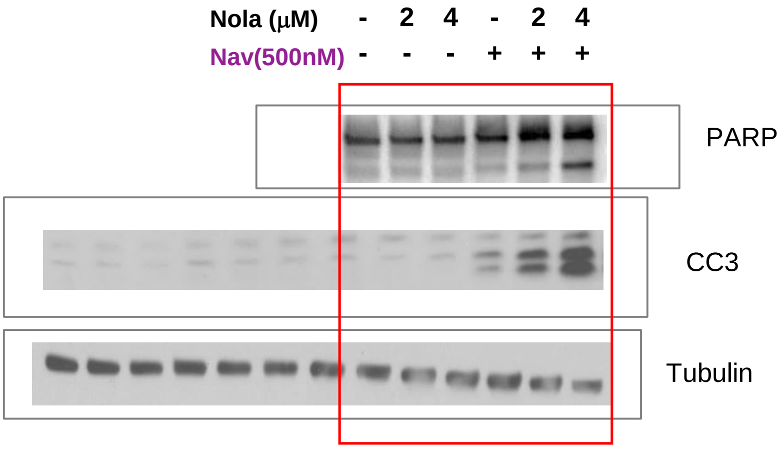

Supplementary Figure S15. Uncropped gels related to Figure 3.

A

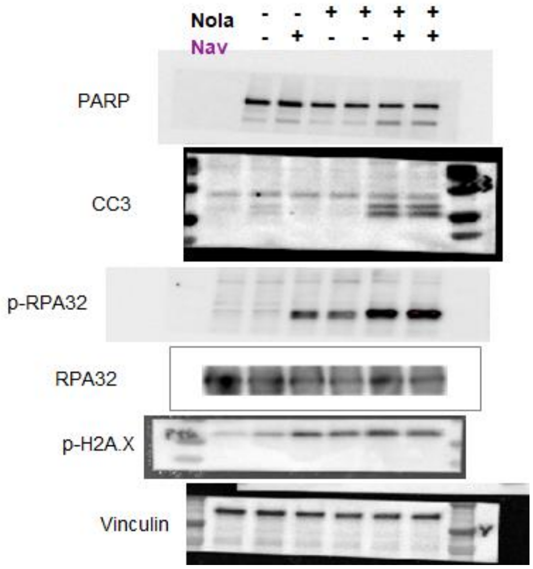

C

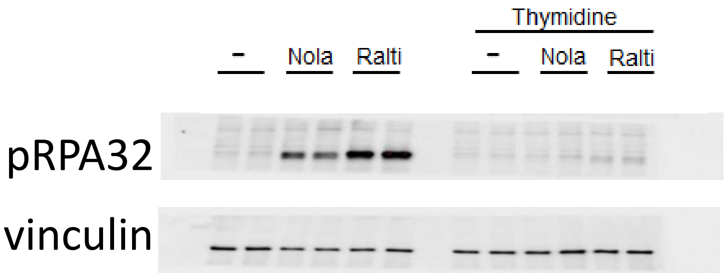

I

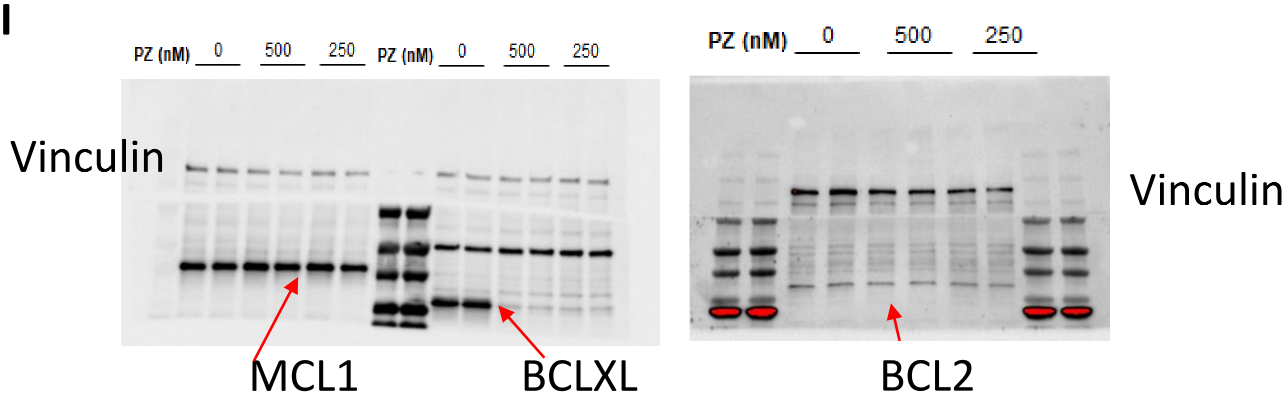

Supplementary Figure S16. Uncropped gels related to Figure 4.

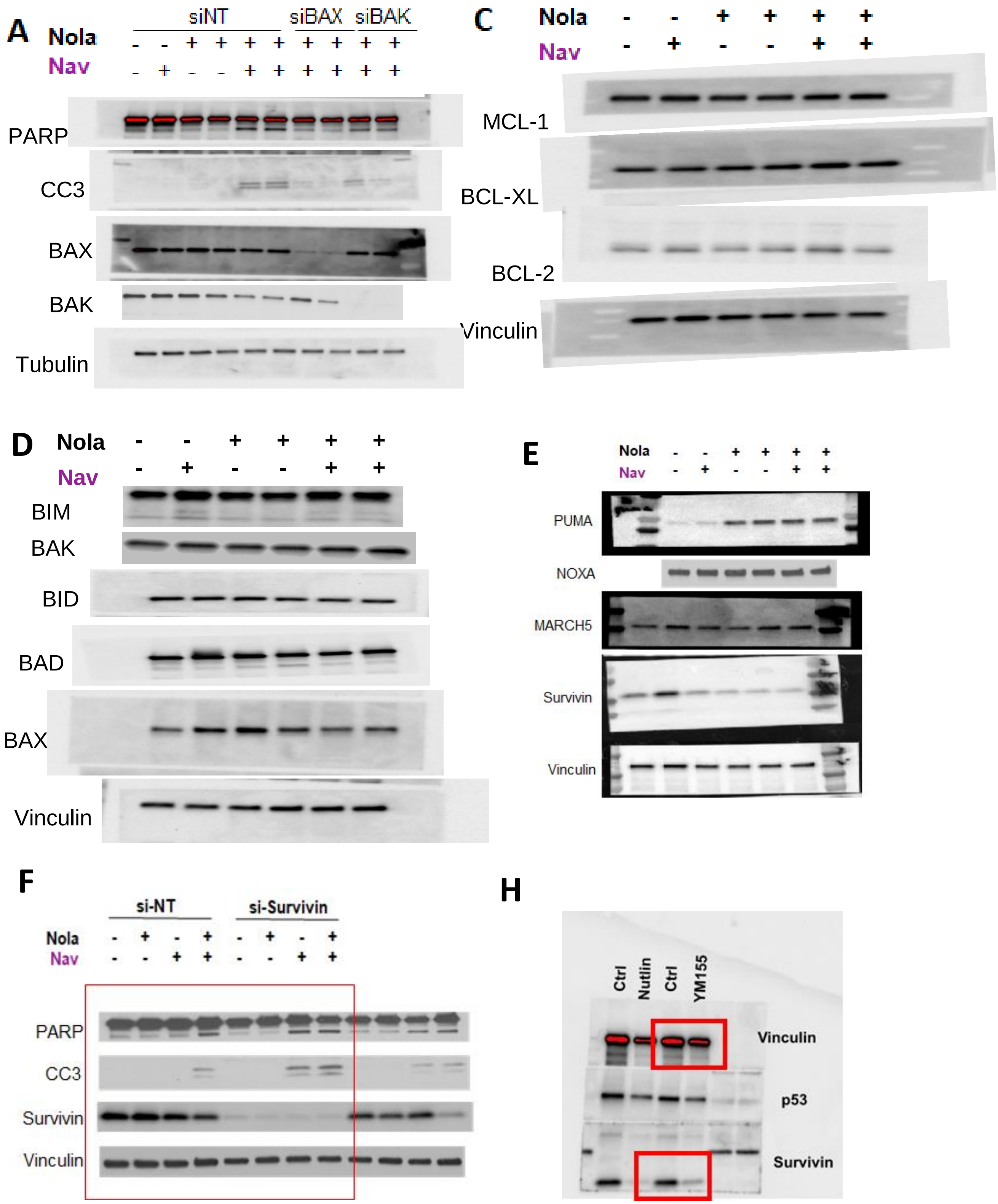

Supplementary Figure S17. Uncropped gels related to Figure 5.

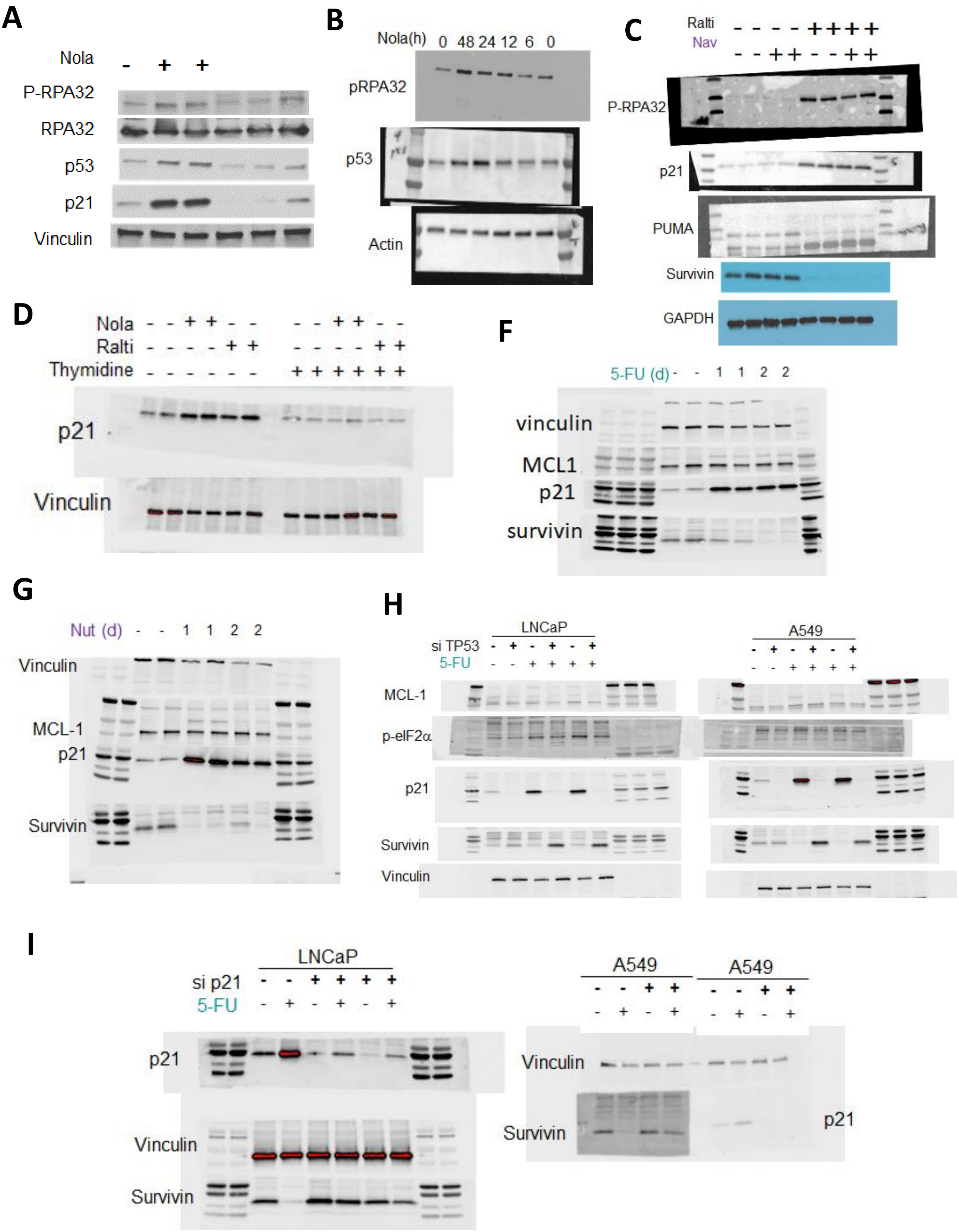

Supplementary Figure S18. Uncropped gels related to Figure 6.

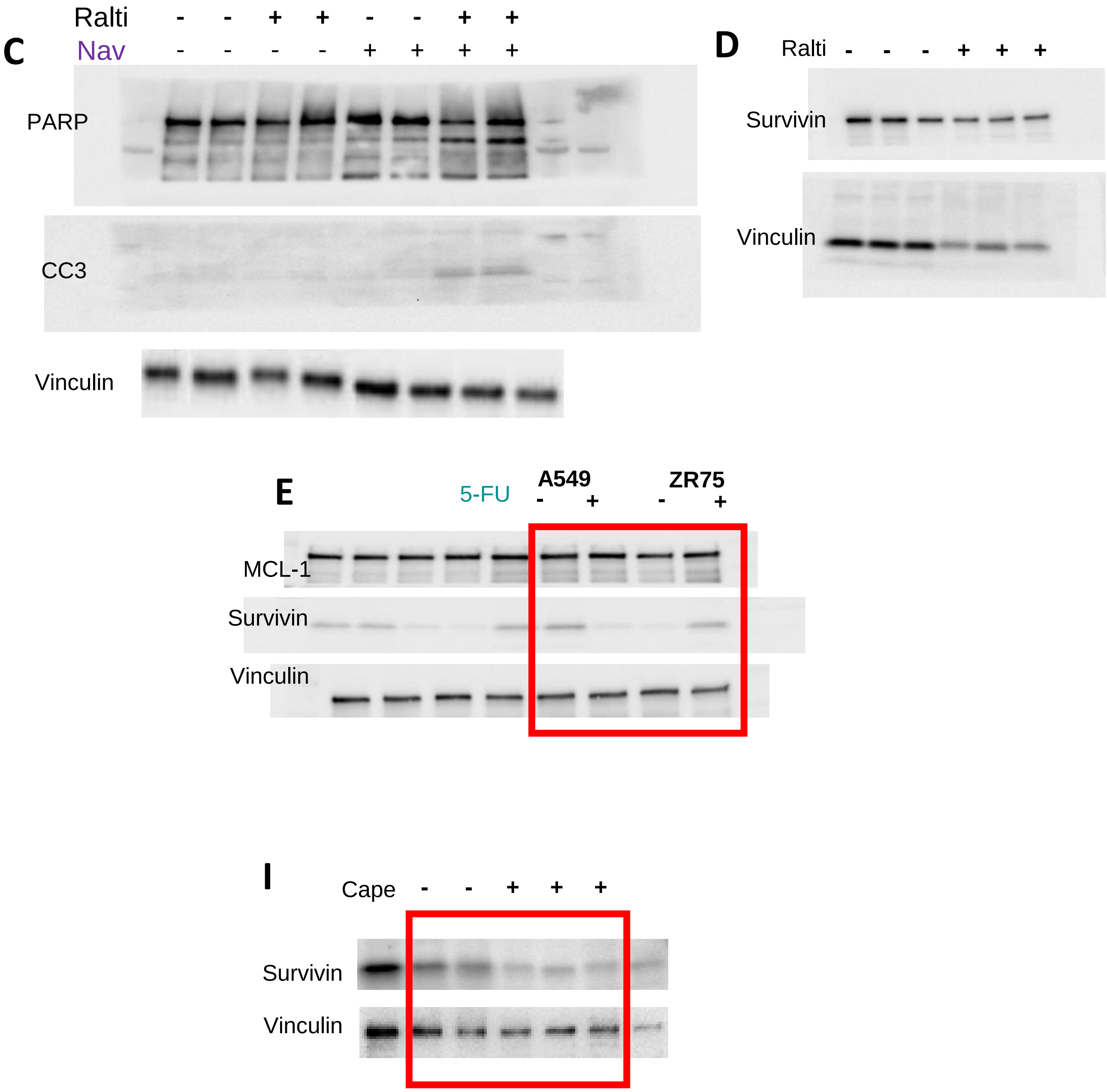

Supplementary Figure S19. Uncropped gels related to Figure 7.

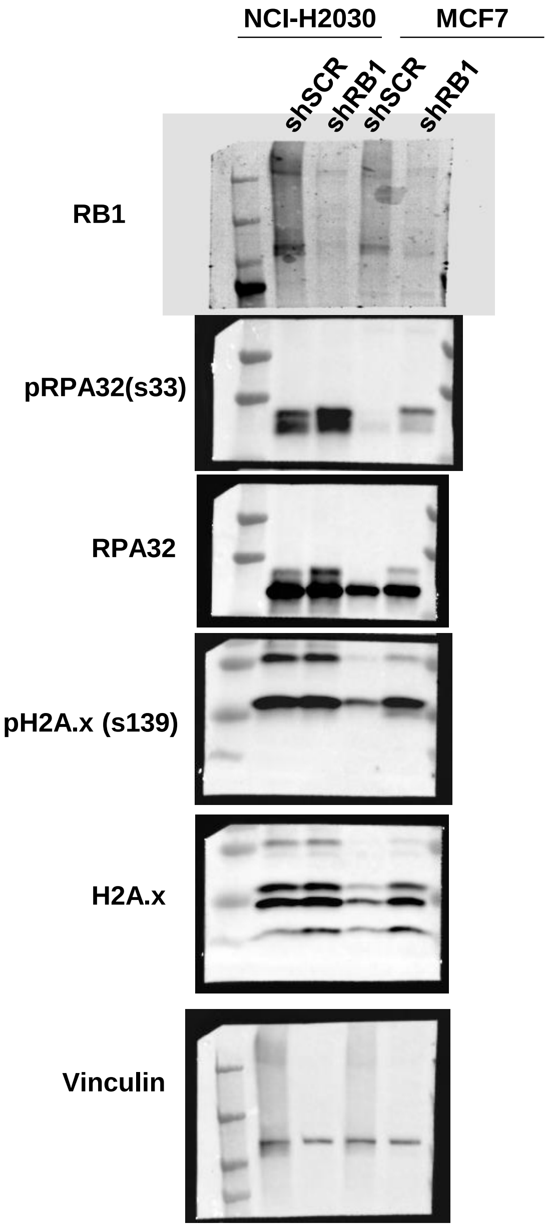

Supplementary Figure S20. Uncropped gels related to Figure S4.

B

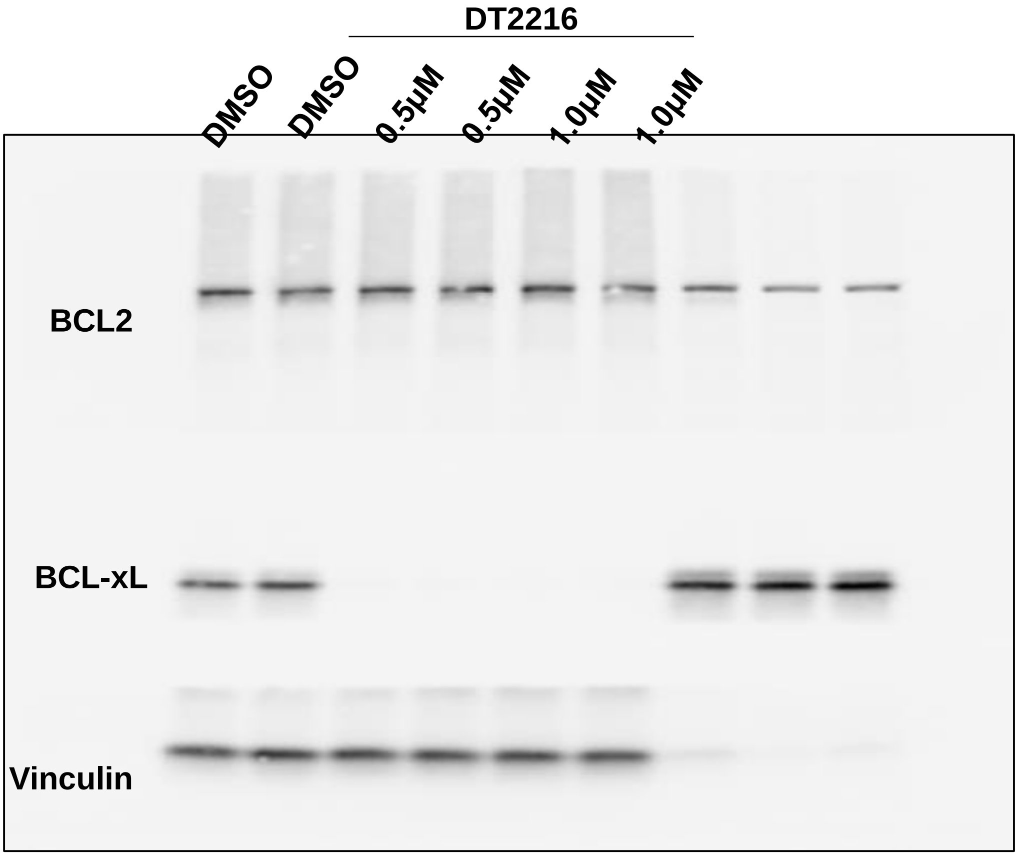

Supplementary Figure S21. Uncropped gels related to Figure S7.

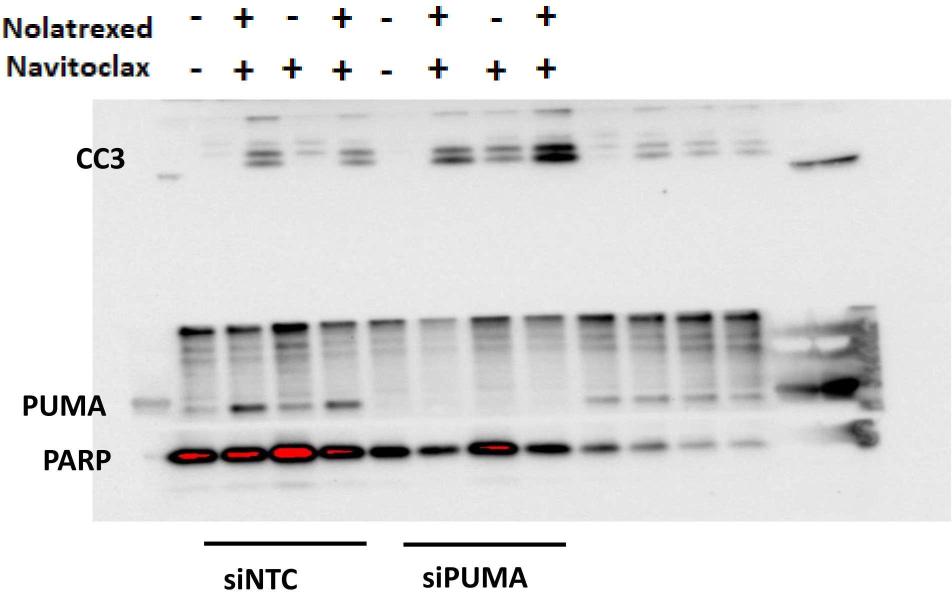

Supplementary Figure S22. Uncropped gels related to Figure S8.

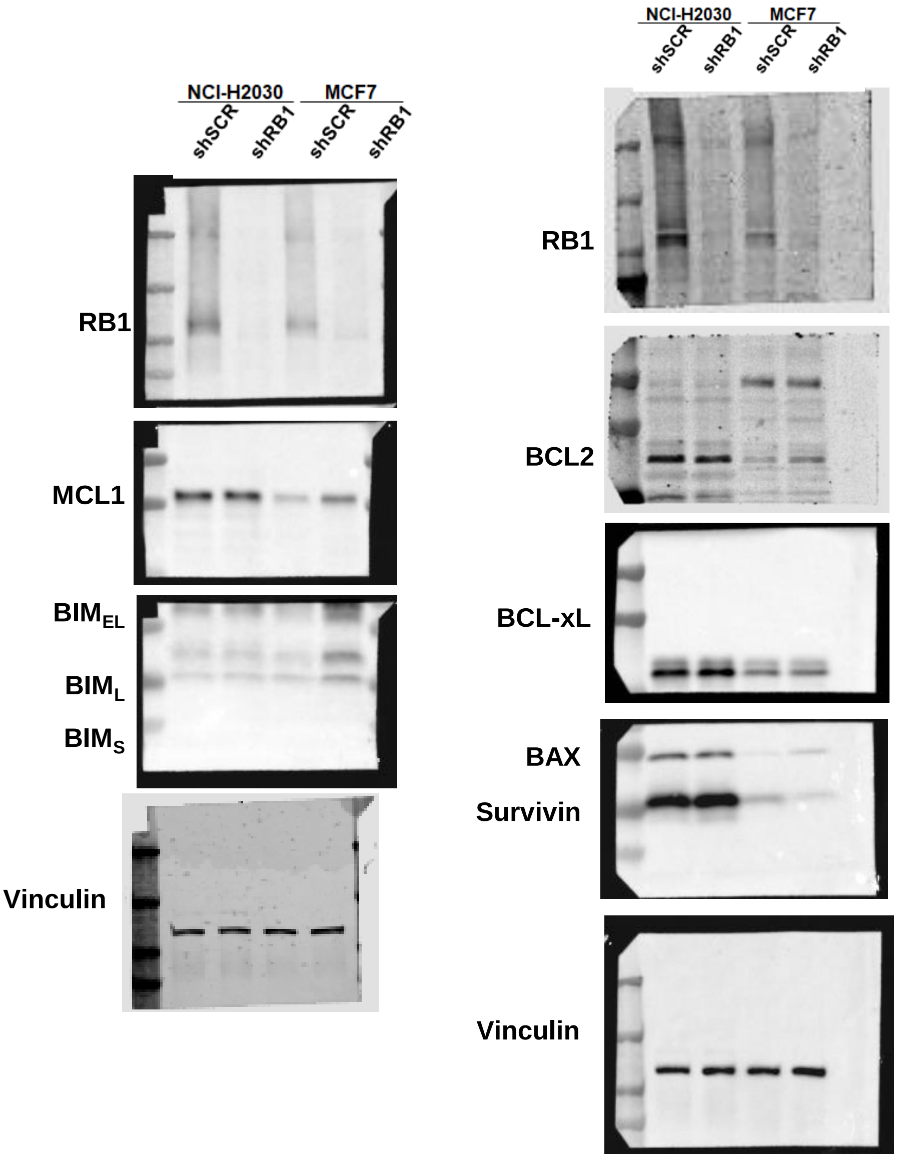

Supplementary Figure S23. Uncropped gels related to Figure S9.

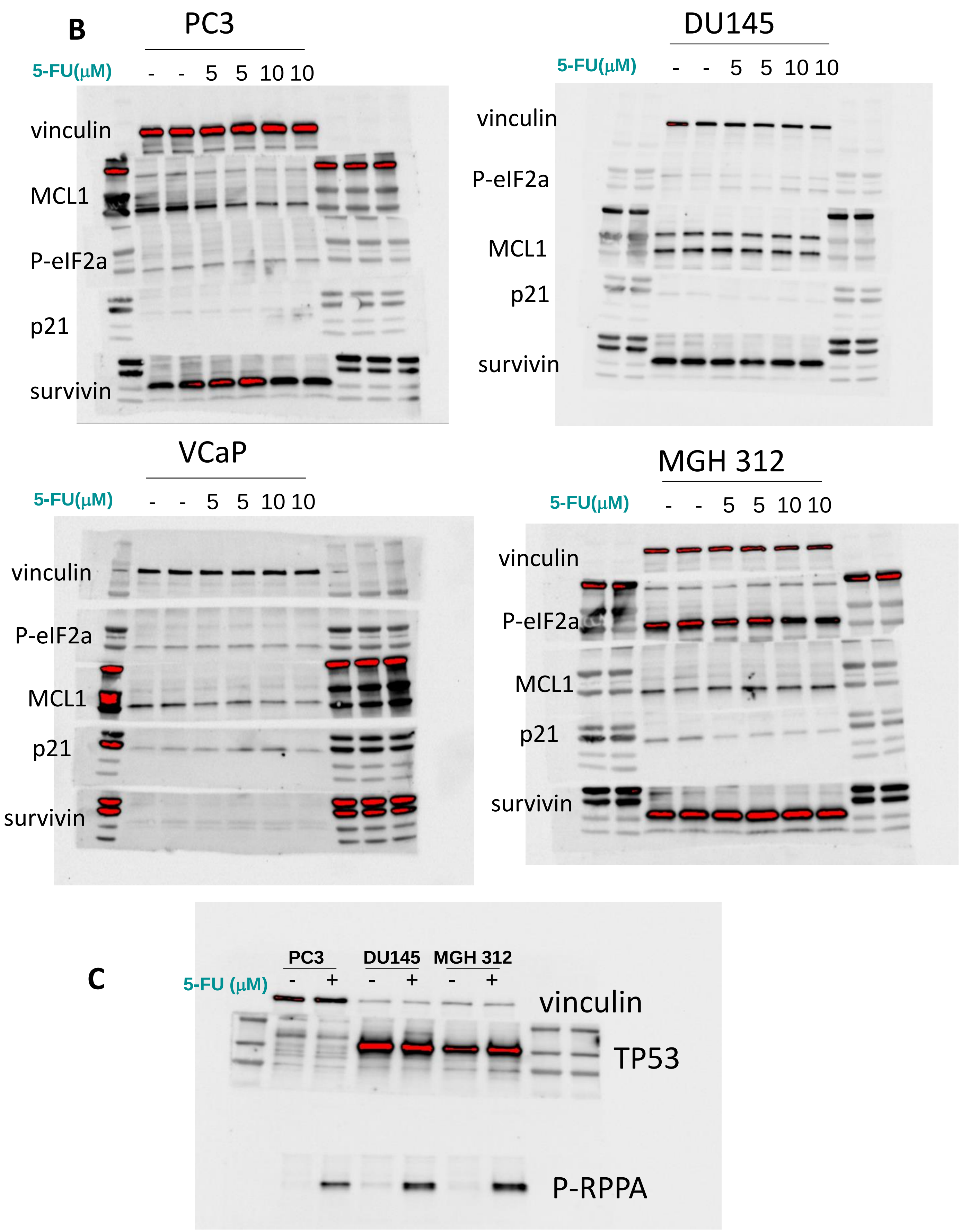

Supplementary Figure S24. Uncropped gels related to Figure S10.

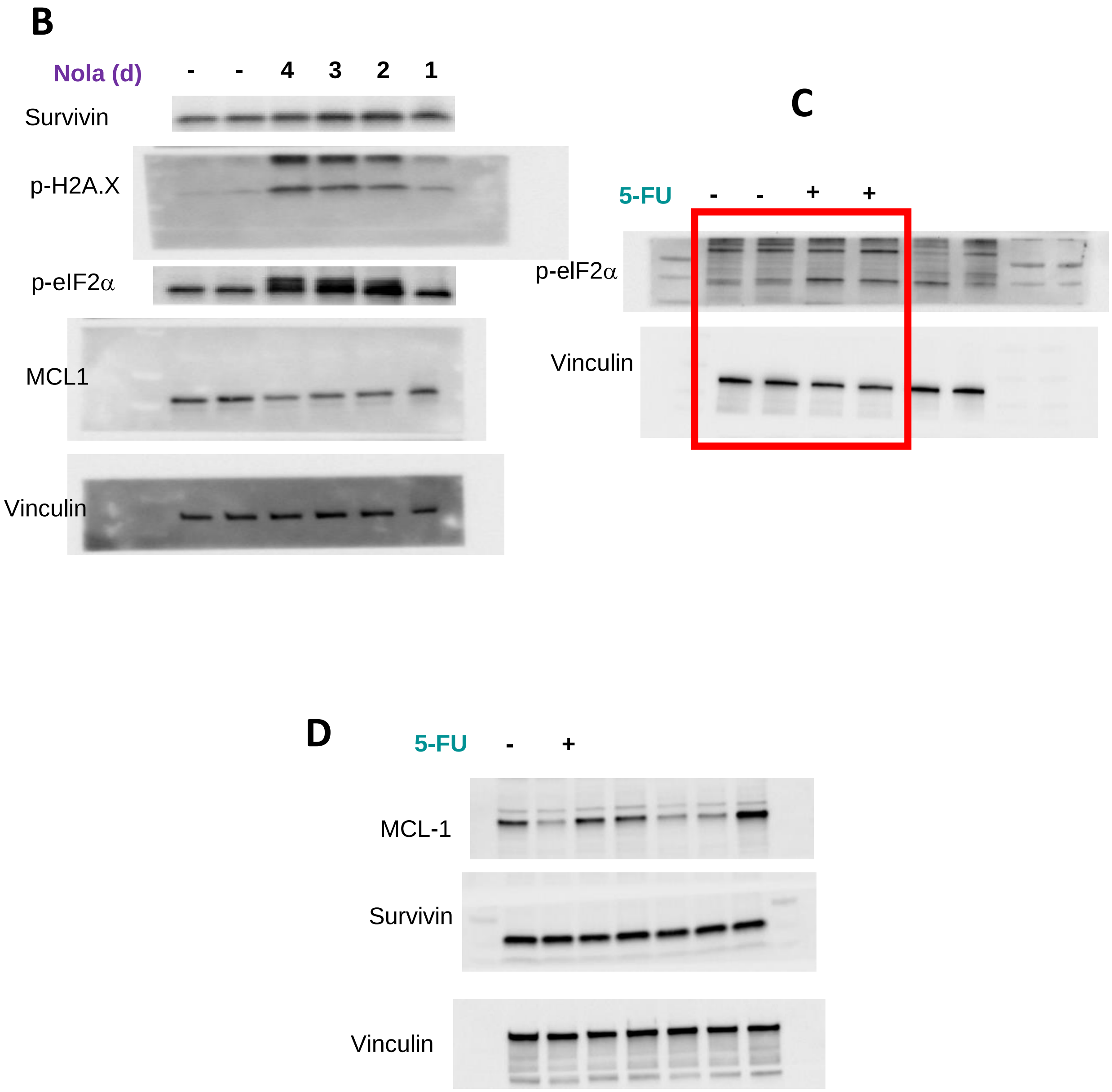

Supplementary Figure S25. Uncropped gels related to Figure S11.
