# Supplementary material for: BH3 mimetics targeting BCL-XL have efficacy in solid tumors with RB1 loss and replication stress
Source: Nat Commun. 2025 May 28;16:4931. doi: 10.1038/s41467-025-60238-x (PMC12119881; doi:10.1038/s41467-025-60238-x)
Supplement: Supplementary file 2 — Reporting Summary [file 41467_2025_60238_MOESM2_ESM.pdf]

nature portfolio

Corresponding author(s): Steven Balk

Last updated by author(s): Mar 10, 2025

Reporting Summary

Nature Portfolio wishes to improve the reproducibility of the work that we publish. This form provides structure for consistency and transparency in reporting. For further information on Nature Portfolio policies, see our Editorial Policies and the Editorial Policy Checklist.

Statistics

For all statistical analyses, confirm that the following items are present in the figure legend, table legend, main text, or Methods section.

n/a	Confirmed	
☐	☒	The exact sample size (n) for each experimental group/condition, given as a discrete number and unit of measurement
☐	☒	A statement on whether measurements were taken from distinct samples or whether the same sample was measured repeatedly
☐	☒	The statistical test(s) used AND whether they are one- or two-sided<br>*Only common tests should be described solely by name; describe more complex techniques in the Methods section.*
☒	☐	A description of all covariates tested
☒	☐	A description of any assumptions or corrections, such as tests of normality and adjustment for multiple comparisons
☒	☐	A full description of the statistical parameters including central tendency (e.g. means) or other basic estimates (e.g. regression coefficient) AND variation (e.g. standard deviation) or associated estimates of uncertainty (e.g. confidence intervals)
☐	☒	For null hypothesis testing, the test statistic (e.g. F, t, r) with confidence intervals, effect sizes, degrees of freedom and P value noted<br>*Give P values as exact values whenever suitable.*
☒	☐	For Bayesian analysis, information on the choice of priors and Markov chain Monte Carlo settings
☒	☐	For hierarchical and complex designs, identification of the appropriate level for tests and full reporting of outcomes
☒	☐	Estimates of effect sizes (e.g. Cohen's d, Pearson's r), indicating how they were calculated

Our web collection on statistics for biologists contains articles on many of the points above.

Software and code

Policy information about availability of computer code

Data collection	no software was used
Data analysis	no software was used

For manuscripts utilizing custom algorithms or software that are central to the research but not yet described in published literature, software must be made available to editors and reviewers. We strongly encourage code deposition in a community repository (e.g. GitHub). See the Nature Portfolio guidelines for submitting code & software for further information.

Data

Policy information about availability of data

All manuscripts must include a data availability statement. This statement should provide the following information, where applicable:

- Accession codes, unique identifiers, or web links for publicly available datasets
- A description of any restrictions on data availability
- For clinical datasets or third party data, please ensure that the statement adheres to our policy

All data have been provided in the manuscript

Research involving human participants, their data, or biological material

Policy information about studies with human participants or human data. See also policy information about sex, gender (identity/presentation), and sexual orientation and race, ethnicity and racism.

Reporting on sex and gender	Tumor materials are all derived from males as only males get prostate cancer.
Reporting on race, ethnicity, or other socially relevant groupings	Tumor materials are collected without information on race or ethnicity
Population characteristics	not applicable
Recruitment	not applicable
Ethics oversight	Beth Israel Deaconess Medical Center IRB

Note that full information on the approval of the study protocol must also be provided in the manuscript.

Field-specific reporting

Please select the one below that is the best fit for your research. If you are not sure, read the appropriate sections before making your selection.

[x] Life sciences [ ] Behavioural & social sciences [ ] Ecological, evolutionary & environmental sciences

For a reference copy of the document with all sections, see nature.com/documents/nr-reporting-summary-flat.pdf

Life sciences study design

All studies must disclose on these points even when the disclosure is negative.

Sample size	sample size was based on estimates of variability in previous studies
Data exclusions	no data were excluded
Replication	results were replicated in at least 3 independent experiments
Randomization	mice were randomized for drug treatment studies
Blinding	tumor size measurements were taken with blinding as to the treatment group

Reporting for specific materials, systems and methods

We require information from authors about some types of materials, experimental systems and methods used in many studies. Here, indicate whether each material, system or method listed is relevant to your study. If you are not sure if a list item applies to your research, read the appropriate section before selecting a response.

Materials & experimental systems

n/a	Involved in the study	
[ ]	[x]	Antibodies
[ ]	[x]	Eukaryotic cell lines
[x]	[ ]	Palaeontology and archaeology
[ ]	[x]	Animals and other organisms
[x]	[ ]	Clinical data
[x]	[ ]	Dual use research of concern
[x]	[ ]	Plants

Methods

n/a	Involved in the study	
[x]	[ ]	ChIP-seq
[x]	[ ]	Flow cytometry
[x]	[ ]	MRI-based neuroimaging

Antibodies

Antibodies used

Cell Signaling Technology 18799S Vinculin (E1E9V) XP® Rabbit mAb (HRP Conjugate), 100 ul
Cell Signaling Technology 4688S cdc25C (5H9) Rabbit mAb #4688
Cell Signaling Technology 9529S Phospho-cdc25C (Ser198) Antibody #9529
Cell Signaling Technology 12028S Phospho-cdc25C (Thr48) (D2H3) Rabbit mAb #12028
Cell signaling 39224S MCL-1 antibody
Cell signaling 8479S Mic1 Rabit antibody

	Cell Signaling 94296S Mcl-1 (D2W9E) Rabbit mAb #94296<br>Thermo fisher MA5-17230 UCHL3 monoclonal antibody<br>Cell Signaling 9309 Rb (4H1) Mouse mAb<br>Santa Cruz sc-102 Rb Antibody (IF8)<br>BD Bioscience 554136 Purified Mouse Anti-Human Retinoblastoma Protein<br>Santa Cruz sc-293185 BRCA2 Antibody (3D12): sc-293185<br>CELL SIGNALING TECHNOLOGY 9718S Phospho-Histone H2A.X (Ser139) (20E3) Rabbit mAb<br>Cell Signaling Technologies 5438S phosphoH2A/X<br>R&D Systems 5461/1 Foxy 5<br>Cell Signaling Technologies 2808 Survivin (71G4B7) Rabbit mAb #2808<br>Cell Signaling Technologies 2764 BCL-XL Rabbit mAb (54H6)<br>Santa Cruz sc-8392 AC Anti-Bcl-xL Antibody (H-5) AC<br>Thermo Fisher MA5-15142 Bcl-xL Monoclonal Antibody (C.85.1)<br>Santa Cruz Biotechnology sc-126 Anti-p53 Antibody (DO-1): sc-126<br>Cell Signaling 2527T P53 antibody<br>Cell Signaling 9721S Phospho-eIF2α (Ser51) Antibody #9721<br>Cell signaling 12450S Puma (D30C10) Rabbit mAb<br>Cell Signaling Technology 88639S ROR2 (D3B6F) Rabbit mAb<br>Santa Cruz Biotechnology sc-6246 p21 Antibody (F-5) 200 µg/ml<br>Cell signaling technology 5453S Mcl-1 (D35A5) Rabbit mAb 100 ul<br>Cell signaling technology 9664S Cleaved Caspase-3 (Asp175) (5A1E) Rabbit mAb #9664 100 uL<br>Cell Signaling Technology 8884S GAPDH (D16H11) XP Rabbit mAb (HRP Conjugate)<br>Cell Signaling Technology 9546S Cleaved PARP (Asp214) (19F4) Mouse mAb (Human Specific)<br>Cell Signaling 9532S PARP (46D11) Rabbit mAb #9532 100 uL<br>Promega W4011 Anti-Rabbit IgG (H+L), HRP Conjugate<br>Promega W4021 Anti-Mouse IgG (H+L), HRP Conjugate
Validation	Antibody validations were based on data from the respective companies.

Eukaryotic cell lines

Policy information about cell lines and Sex and Gender in Research

Cell line source(s)	Cells were purchased from ATCC and maintained in RPMI 1640 with L-Glutamine (Corning, #MT10040CV) or DMEM with L-Glutamine and 4.5 g/L and Sodium Pyruvate (Corning, #MT10013CV), both supplemented with 10% fetal bovine serum (Gibco, #A3160401). The CaPan-1 and CaPan-1BRCA2 isogenic lines were a kind gift from the lab of Erika T. Brown, and maintained in DMEM supplemented with 20% fetal bovine serum.
Authentication	Cell lines were generally employed for less than 25 passages before fresh stocks were thawed, and cell identity was confirmed by STR profiling (ATCC) for cells passaged longer.
Mycoplasma contamination	Cells were tested monthly for Mycoplasma using the MycoAlert Kit (Lonza, ##LT07-218) according to the manufacturer's instructions.
Commonly misidentified lines (See ICLAC register)	none

Animals and other research organisms

Policy information about studies involving animals; ARRIVE guidelines recommended for reporting animal research, and Sex and Gender in Research

Laboratory animals	6-8 week old ICRSC-M, IcrTac:ICR-Prkdcscid mice (Taconic)
Wild animals	none
Reporting on sex	males were used as prostate cancer is confined to males
Field-collected samples	none
Ethics oversight	Beth Israel Deaconess IACUC

Note that full information on the approval of the study protocol must also be provided in the manuscript.

Plants

Seed stocks	none
Novel plant genotypes	none
Authentication	none
